# Enhanced Anti‐Aging Properties of Silymarin With a Natural Phytochemical Ratio Over Commercial Formulations

**DOI:** 10.1111/jocd.70780

**Published:** 2026-03-05

**Authors:** Sen Hou, Anning Wang, Hanliang Song, Anzhang Li

**Affiliations:** ^1^ Guyu Biotechnology Group Company Ltd. Guangzhou Guangdong China; ^2^ Guangzhou Qingnang Biotechnology Company Ltd. Guangzhou Guangdong China

**Keywords:** anti‐aging, milk thistle, silybin, silychristin, silydianin, silymarin

## Abstract

**Background:**

Silymarin, a bioactive mixture widely used in anti‐aging cosmetic products, is primarily composed of silybin, silychristin, and silydianin. The relative proportions of these three compounds in milk thistle seeds, the natural source of silymarin, differ significantly from those found in commercially available silymarin formulations. Notably, under natural conditions, silymarin contains a higher proportion of silybin than its commercial formulations.

**Objectives:**

This study aimed to comparatively evaluate how differences in these ratios affect the anti‐aging properties of silymarin and to unveil the underlying mechanism.

**Methods:**

We quantified the natural and commercial ratio of silymarin. We measured the cytotoxicity of the natural and commercial silymarin mixtures, as well as individual silybin, silychristin, and silydianin. We studied the matrix metalloproteinases (MMPs) inhibition capacities of natural and commercial silymarin mixtures as a sign of their anti‐aging properties. The mechanism of enhanced anti‐aging properties was studied by ROS scavenging assay, reducing power assay, pro‐inflammatory factor inhibition assay, using natural and commercial silymarins, as well as individual silybin, silychristin, and silydianin. Finally, an anti‐wrinkle experiment on human faces was conducted using an emulsion containing natural silymarin.

**Results:**

We demonstrated that silymarin composed of the natural phytochemical ratio exhibits superior anti‐aging efficacy compared to silymarin prepared using the “commercial ratio.” This finding was evidenced by its greater inhibitory effect on MMPs in human dermal fibroblasts, which are involved in collagen degradation in the dermis. Thus, the natural silymarin mixture was more effective at preventing collagen degradation. Reactive oxygen species (ROS) production, the primary trigger for MMP synthesis, was attenuated more strongly by the natural silymarin mixture, likely due to the higher ROS scavenging capacity of silybin compared to silychristin and silydianin. Notably, although the reducing ability of individual compounds (silybin, silychristin, and silydianin), as well as both natural and commercial silymarin mixtures, was studied, no correlation between reducing power and ROS scavenging capacity was observed. The natural silymarin mixture was also more effective at downregulating tumor necrosis factor‐alpha (TNF‐α), a key pro‐inflammatory cytokine, though it did not affect interleukin‐6 expression. This effect was also attributed to the higher anti‐TNF‐α capacity of silybin compared to that of silychristin and silydianin. Finally, an emulsion containing the natural ratio of silymarin was found to be effective in reducing facial wrinkles.

**Conclusion:**

To our knowledge, this study is the first to unveil the mechanistic basis underlying the superior anti‐aging properties of silymarin containing a natural phytochemical ratio over commercial silymarin formulations. These findings are anticipated to be useful in producing better silymarin‐based anti‐aging cosmetic products.

## 1 Introduction

Silymarin, which is primarily composed of flavonolignans—including silybin, silychristin, and silydianin—is widely utilized as an anti‐aging agent in the cosmetics industry [1, 2]. However, optimizing the ratio of these compounds is crucial for enhancing the efficacy of silymarin in cosmetic applications. Currently, silymarin is extracted from the seeds of *Silybum marianum* (milk thistle) without any chemical modification [3]. However, during the purification process, differences in extraction efficiencies among the individual components of silymarin can result in altered proportions of silybin, silychristin, and silydianin. Therefore, the ratios of these compounds differ considerably between commercial silymarin formulations and natural milk thistle seeds. Notably, the ratio of silybin, silychristin, and silydianin in milk thistle seeds (hereafter called the “natural ratio”) has been determined over a long course of evolution. The *Silybum marianum* plant has optimized the production of these compounds during evolution to survive adverse conditions [4], including extreme temperatures, heavy metal exposure, and irradiation [5]. Interestingly, the evidence suggests that reactive oxygen species (ROS) production serves as a key shared mechanism through which plants support their growth in adverse environments [6]. Under these conditions, plants exhibit a broad range of physiological responses, including the modulation of protective phytochemicals. As a result, the natural ratio of flavonolignans in wild plants may represent an evolutionary strategy that confers maximal resistance to environmental challenges.

The anti‐aging efficacy of silymarin is largely attributed to its ability to prevent collagen degradation in the skin. Photoaging, characterized by wrinkles, sagging, and loss of skin elasticity, is primarily induced by ultraviolet (UV) radiation, which reduces collagen synthesis and enhances collagen breakdown in the dermis [7]. UVA is the most penetrative and damaging of all UV rays, causing decreased collagen production in fibroblasts and increasing collagen degradation [8]. Notably, matrix metalloproteinases (MMPs), which play a key role in collagen degradation, are upregulated by the ROS generated in response to UV exposure [9]. Therefore, the effective scavenging of ROS and the subsequent inhibition of MMP activity are considered two fundamental functional properties of effective anti‐aging ingredients [10, 11].

Initially, silymarin was used as a natural medicine for the treatment of liver diseases, such as hepatitis and cirrhosis [12, 13]. However, accumulating evidence regarding the anti‐aging efficacy of silymarin demonstrated its value as a promising cosmetic ingredient [14]. In addition to silybin, silychristin, and silydianin, silymarin has also been found to contain other ingredients, such as isosilybin, 2,3‐dehydrosilybin, and taxifolin [15]. Nevertheless, silybin, silychristin, and silydianin still account for the majority of active components in silymarin. Silybin is known to exist in two diastereoisomeric forms, silybin A and silybin B [16]. Although silybin, silychristin, and silydianin share the same molecular formula (C_25_H_22_O_10_), they show varying biological properties [15].

Evidence shows that silybin, silychristin, and silydianin act as free radical scavengers, countering the ROS induced due to environmental stress and supporting the growth of *Silybum marianum* . This ROS scavenging capacity is also crucial for the anti‐aging effects of silymarin. Hence, the relative ratio of silybin, silychristin, and silydianin in silymarin influences its anti‐aging properties. However, as mentioned previously, the *Silybum marianum* plant has optimized the ratio of these compounds to scavenge ROS and ensure its survival. Given the role of ROS scavenging as an anti‐aging mechanism, it follows that the natural ratio of silybin, silychristin, and silydianin in silymarin would provide the most effective anti‐aging benefits. However, due to loss during extraction and purification, commercial silymarin formulations contain lower amounts and different ratios of silybin, silychristin, and silydianin when compared to milk thistle seeds. Although these extraction efficiencies are expected to improve with technological advancements, resulting in commercial formulations with a flavonolignan composition similar to that of natural milk thistle seeds, the “commercial ratio” remains different from the “natural ratio” at present. However, the influence of the phytochemical ratio on the anti‐aging functions of silymarin is yet to be explored in detail.

In the study, we compared the anti‐aging properties of silymarin containing a natural phytochemical ratio (natural silymarin mixtures) to those of a commercial silymarin formulation. We studied the MMP inhibition, ROS scavenging, and ABTS scavenging capacities of these mixtures, as well as those of pure silybin, silychristin, and silydianin alone. The inhibition capacities of the two silymarin mixtures on the pro‐inflammatory factors tumor necrosis factor (TNF)‐α and interleukin (IL)‐6 were also examined. Finally, the mechanisms underlying ratio‐related functional differences were explored, and the anti‐wrinkle efficacy of a topical formulation containing the natural silymarin mixture was tested on human facial skin.

## 2 Materials and Methods

### 2.1 Materials and Cells

Samples of silymarin and milk thistle seeds were collected from different manufacturers in China. Silymarin samples were purchased from Givaudan SA, Switzerland (Silymarin A); StarHealth Botanical Technology Company Ltd., Beijing, China (Silymarin B); Organic Herb Company Ltd., Hunan, China (Silymarin C); Hanzhong TRG Biotech Company Ltd., Shanxi, China (Silymarin D); and Panjin Tianyuan Pharmaceutical Company Ltd., Panjin, China (Silymarin E), respectively. The milk thistle seeds were purchased from StarHealth Botanical Technology Company Ltd., Beijing, China (Milk Thistle Seed A); Organic Herb Company Ltd., Hunan, China (Milk Thistle Seed B); Hanzhong TRG Biotech Company Ltd., Shanxi, China (Milk Thistle Seed C); and Bozhou Qianwengtang Trading Company Ltd., Haozhou, China (Milk Thistle Seed D), respectively. In industrial production, the silymarin samples were extracted from the milk thistle seeds without chemical or biological reactions. Human skin fibroblast (HSF) cells were obtained from Fenghui Biotechnology Company Ltd., China. These HSF cells were cultured in Dulbecco's Modified Eagle Medium (DMEM) containing 10% fetal bovine serum (FBS; Gibco, USA) and 1% penicillin/streptomycin (100 U/mL–100 μg/mL) under standard conditions.

### 2.2 High‐Performance Liquid Chromatography (HPLC)

Milk thistle seeds were ground into powder. Both silymarin and milk thistle powders were mixed with 95% alcohol, ultrasonicated for 20 min, filtered with a 0.22 μm filter membrane, and then loaded on HPLC for analysis. All product samples and peak fractions were analyzed using HPLC on an Agilent Infinite 1260I system equipped with a C18 column (250 × 4.6 mm, 5 μm; Agilent ZORBAX). Solvent A (methanol; chromatographically pure) and solvent B (water +0.1% methanol) were used as the mobile phases. The elution was performed using the following gradient program: 0–10 min, 40% A; 10–13 min, 40%–60% A; 13–14 min, 60% A; 14–22 min, 60%–40% A. Elution was stopped after 22 min. Moreover, the flow rate was 0.9 mL/min; injection volume, 10 μL; detection wavelength, 288 nm; and column temperature, 30°C. Samples were quantified by comparing the peak areas with those of corresponding standards. The chromatographic peaks of silybin, silychristin, and silydianin were identified by matching their retention times and UV spectra against those of reference compounds.

### 2.3 Cell Viability Assay

HSF cells were seeded in 96‐well plates at a density of 0.8 × 10^5^ cells/cm^2^ and cultured for 24 h. Then, the cells were incubated with 100 μL of cell culture medium containing different concentrations of a natural silymarin mixture, commercial silymarin formulation, silybin alone, silychristin alone, or silydianin alone (0.00001%, 0.00005%, 0.0001%, 0.0005%, 0.001%, and 0.005% w/w) at 37°C for 24 h. Then, the cell viability was assessed using the MTT assay. For the MTT assay, the medium in each well was replaced with 200 μL of DMEM containing 0.5 mg/mL MTT, and the cells were incubated in the dark at 37°C for 2 h. Following incubation, the medium in each well was replaced with 200 μL of dimethyl sulfoxide (DMSO), and the optical density (OD) values were measured at 490 nm using a microplate reader (Epoch2, BioTek, USA). Untreated cells served as the blank control (BC). The cell viability (V) was measured using (Equation 1), as follows:

(1) $$V=\frac{\mathrm{OD}-{\mathrm{OD}}_{0}}{{\mathrm{OD}}_{s}-{\mathrm{OD}}_{0}}\times 100\%$$

where OD represents the absorbance value of each test sample, OD_0_ represents the absorbance value of the blank well, and OD_s_ represents the absorbance value of the solution alone (without cells). All measurements were repeated three times. The results were presented as the mean ± standard deviation (SD) of the three replicates.

### 2.4 MMP‐1 Expression Assay

The mRNA expression of MMP‐1 was measured via quantitative real‐time PCR (qPCR). First, HSF cells were seeded in 12‐well plates at a density of 1 × 10^5^ cells/cm^2^ and cultured for 24 h. Then, the cells were incubated in phosphate‐buffered saline (PBS) and exposed to UVA irradiation (20 J/cm^2^) with a UV aging test chamber (Beijing Pulinsaisi Technology Company Ltd., China). Subsequently, the cells were incubated with DMEM containing different concentrations of the natural silymarin mixture and commercial silymarin formulation (0.00005%, 0.0005%, and 0.005% w/w) for an additional 24 h. After treatment, the cells were rinsed with PBS and lysed to extract total RNA using the RNA‐easy Isolation Reagent (Vazyme Biotech, China) according to the manufacturer's instructions. Then, cDNA was generated using the HiScript II Q RT SuperMix for qPCR (Vazyme Biotech, China). Finally, qPCR was performed using the ChamQ Universal SYBR qPCR Master Mix (Vazyme Biotech, China) on a CFX Opus 96 real‐time PCR system (Bio‐Rad, USA) equipped with Bio‐Rad CFX Maestro software. The relative expression of all target genes was normalized based on the expression of the internal reference β‐actin. Untreated cells served as the BC group, while those treated with UVA irradiation alone served as the negative control group (NC). All measurements were repeated three times. The results were presented as the mean ± SD. All the primer sequences used for qPCR are shown in Table 1.

**TABLE 1:** Primer sequences used for qPCR.

Primer	Sequence
MMP‐1‐F	5′‐ATTCTACTGATATCGGGGCTT‐3′
MMP‐1‐R	5′‐ATGTCCTTGGGGTATCCGTGTAG‐3′
Hum‐β‐Actin‐F	5′‐GTCCACCTTCCAGCAGATGT‐3′
Hum‐β‐Actin‐R	5′‐GTCACCTTCACCGTTCCAGT‐3′

### 2.5 ROS Scavenging Assay

HSF cells were seeded in 96‐well plates at a density of 0.8 × 10^5^ cells/cm^2^ and cultured for 24 h. The cell culture medium was replaced with PBS, and the cells were subsequently exposed to UVA irradiation (15 J/cm^2^). Following this, the cells were incubated with DMEM containing different concentrations of the natural silymarin mixture, commercial silymarin formulation, silybin, silychristin, or silydianin (0.00005%, 0.0005%, and 0.005% w/w) for 1 h. After incubation, the cell culture medium was removed, and the ROS levels were measured using a ROS Assay Kit (Beyotime, China) according to the manufacturer's instructions. The fluorescence intensity in each well was measured at an excitation wavelength of 488 nm and an emission wavelength of 525 nm using a microplate reader (Biotek, USA) equipped with Gen5 software. Untreated cells were considered the BC group, while those exposed to UVA irradiation alone served as the NC group, and those treated with 0.3 mg/mL vitamin C following UVA irradiation served as the positive control (PC) group. All measurements were repeated three times. The results were presented as the mean ± SD.

### 2.6 ABTS Free Radical Scavenging Assay

A working solution was prepared by mixing 7 mmol/L 2, 2′‐azino‐bis(3‐ethylbenzothiazoline‐6‐sulfonic acid) (ABTS) with a 2.45 mmol/L potassium persulfate solution at a 1:1 (v/v) ratio. This mixture was incubated in the dark for 12 h and diluted with water until the OD value at 734 nm reached 0.75 ± 0.05 to obtain the ABTS working solution. The ABTS working solution was mixed with different concentrations of natural silymarin, commercial silymarin, silybin, silychristin, or silydianin (0.0005%, 0.00125%, 0.0025%, 0.005%, 0.0125%, 0.025%, and 0.05% w/w). The OD values of these mixtures were measured at a wavelength of 734 nm using a microplate reader (Biotek, USA) equipped with Gen5 software. The ABTS scavenging capacity (*A*) was calculated using (Equation 2).

(2) $$A=\left(1-\frac{T-T_{0}}{C-C_{0}}\right)\times 100\%$$

Here, T represents the OD of sample solutions containing the ABTS working solution; T_0_ is the OD of sample solutions without any ABTS working solution; C is the OD of reagent mixtures containing the ABTS working solution; and C_0_ is the OD of reagent mixtures without any ABTS working solution. Vitamin C was used as a control (see ABTS removal data by vitamin C in Figure S1). All measurements were repeated three times. The results were presented as the mean ± SD. A baseline‐category logit model was employed to assess the IC50 value for ABTS scavenging (R^2^ > 0.9).

### 2.7 Evaluation of Pro‐Inflammatory Factor Expression

For the enzyme‐linked immunosorbent assay (ELISA), RAW264.7 cells were seeded in 48‐well plates at a density of 1.2 × 10^5^ cells/cm^2^ and cultured for 24 h. Then, these cells were incubated in DMEM containing different concentrations of natural silymarin, commercial silymarin, silybin, silychristin, or silydianin (0.00005%, 0.0005%, and 0.005% w/w)—along with 1 mg/L lipopolysaccharide (LPS)—for 24 h. Finally, the cell culture medium was collected and centrifuged at 1,000 × *g* for 20 min. The relative concentrations of IL‐6 and TNF‐α in the supernatant were measured using the Mouse IL‐6 (Interleukin 6) ELISA Kit and Mouse TNF‐α (Tumor Necrosis Factor Alpha) ELISA Kit (Sangon Biotech, China), respectively, according to the manufacturer's instructions. Untreated cells served as the BC group; those treated with LPS alone served as the NC group; and those treated with 400 mg/L dexamethasone (DXM) and LPS served as the PC group. All measurements were repeated three times. The results were presented as the mean ± SD.

### 2.8 Evaluation of the in Vivo Anti‐Aging Efficacy of Natural Silymarin

All experimental protocols adhered to the Declaration of Helsinki. All participants provided written informed consent, and their privacy rights were strictly maintained. The trial was conducted from June 25, 2025, to July 23, 2025.

Data were obtained from a total of 30 volunteers (3 men and 27 women) aged 31–54 years. Exclusion criteria were as follows: Presence of skin disease, sensitivity to sunlight, current use of medicines or foods known to cause photosensitivity, lesions at the measurement sites, any form of phototherapy within the preceding 6 months, and pregnancy. An emulsion formulation containing the natural silymarin mixture was prepared (detailed formula provided in the Supporting Information Table S1) and applied on the left side of the face, twice daily for 28 days. As a control, a blank sebum formulation, identical in composition but without silymarin, was applied to the right side of the face. The wrinkle area ratio was measured using the VISIA7 imaging system (Canfield, USA) equipped with Image Prof Plus software. Data were collected at baseline (before treatment) and on the 28th day following the twice‐daily application of the test formulation.

### 2.9 Statistical Analysis

Statistical analyses were conducted using GraphPad Prism 5 for Windows. Comparisons between experimental groups were performed using a two‐tailed Student's *t*‐test. The significance thresholds were designated as follows: *p < 0.05, **p < 0.01, ***p < 0.001, and ****p < 0.0001.

## 3 Results and Discussion

**FIGURE 1:**
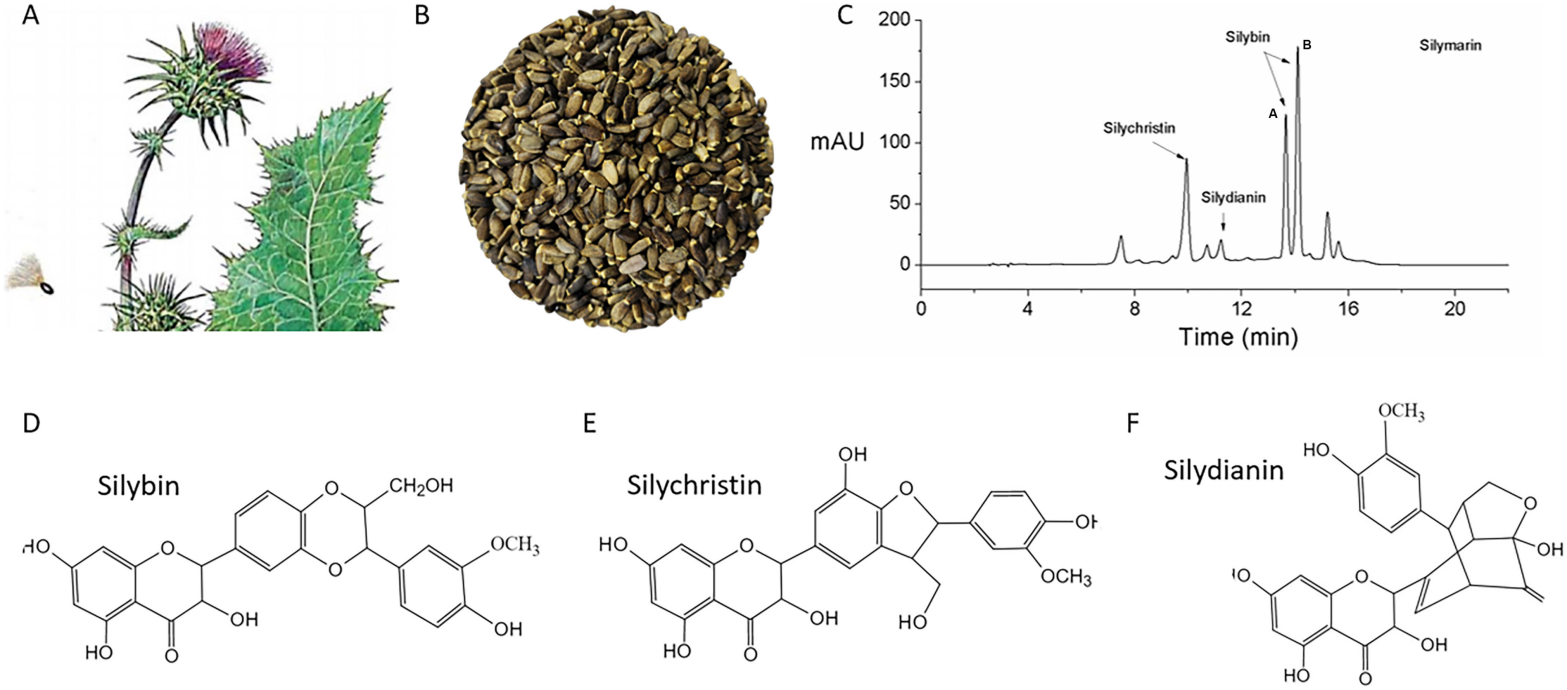
Major components of silymarin. (A) Image showing milk thistle. (B) Image showing milk thistle seeds, from which silymarin is extracted. (C) HPLC data showing the major components of silymarin. (D–F) Chemical structures of silybin (D), silychristin (E), and silydianin (F).

Silymarin, extracted from milk thistle seed, can be used as an ingredient in cosmetic products (Figure 1). Silymarin is primarily composed of silybin, silychristin, and silydianin, which are isomers of each other. Silymarin is also composed of other flavonolignans, such as isosilybin, taxifolin, and 2,3‐dehydrosilybin [17]. Milk thistle seeds, the raw material for silymarin, contain other basic components of living organisms, such as proteins (25%–30%), fixed oil (20%–30%), etc. [17]. On HPLC, silybin shows two peaks corresponding to its diastereoisomeric forms, silybin A and silybin B. The ratio of silybin A to silybin B ranges from 1.2 to 1.5 and remains largely constant across all silybin and silymarin samples. In the present study, silybin A and silybin B were collectively referred to as silybin, in line with previous studies, where silybin A and silybin B were collectively used to study the bioactivities of silybin [18].

In the present study, the HPLC graph revealed that silybin was the most abundant component of silymarin, followed by silychristin and silydianin. Previous reports show that the bioactivities of silybin, silychristin, and silydianin differ from each other. For example, the DPPH free radical scavenging capacity is the highest in silychristin, followed by silydianin and silybin [18]. Therefore, the ratio of silybin, silychristin, and silydianin determines the bioactivity of silymarin.

**FIGURE 2:**
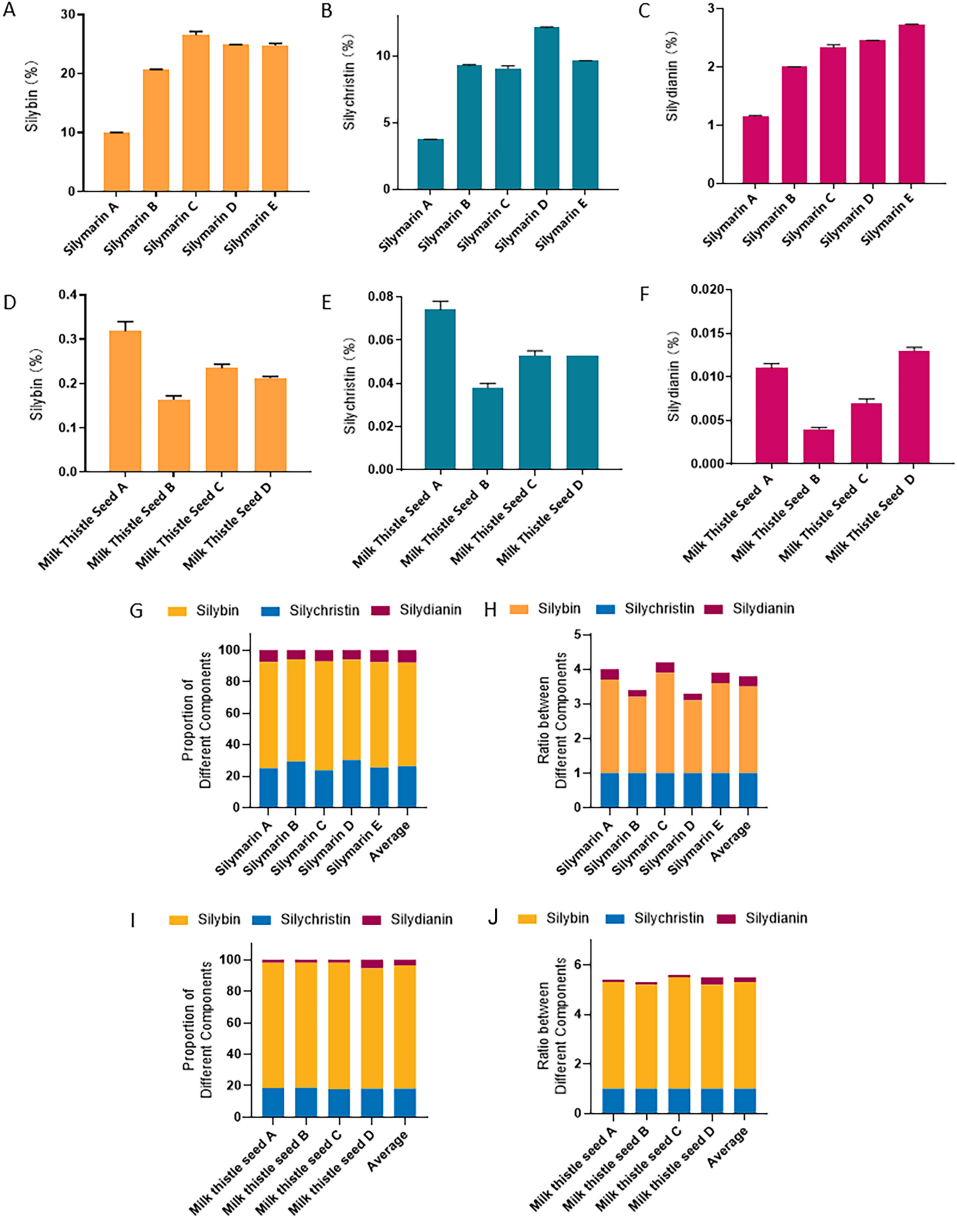
Concentrations of silybin, silychristin, and silydianin in commercial silymarin and milk thistle seeds. (A–C) Concentrations of silybin (A), silychristin (B), and silydianin (C) in commercial silymarin samples. (D–F) Concentrations of silybin (D), silychristin (E), and silydianin (F) in milk thistle seed samples. (G, H) Proportion of different components (G) and their relative ratio (H) in commercial silymarin samples. (I, J) Proportion of different components (I) and their relative ratio (J) in milk thistle seed samples.

In this study, we measured the concentrations of silybin, silychristin, and silydianin in different commercially available silymarin samples (Figure 2). The qualities of the commercially available silymarin formulations, characterized by the concentrations of these flavonolignans, exhibited considerable variations. For instance, the concentration of silybin in silymarin varied from ca. 10% to ca. 25%. Meanwhile, silymarin was also extracted from milk thistle seeds. The concentrations of silybin, silychristin, and silydianin in different milk thistle seeds also differed considerably. For example, the concentration of silybin in milk thistle seed A was twice as high as that in milk thistle seed B. These findings partially explain why silymarin formulations produced by different manufacturers have different phytochemical concentrations.

Interestingly, although the concentrations of silybin, silychristin, and silydianin varied across different commercial silymarin samples, the ratios of these compounds in these samples remained largely consistent. On average, the ratio of silybin, silychristin, and silydianin in commercially available silymarin samples was roughly 2.5:1:0.3. Therefore, this was defined as the commercial ratio of phytochemicals in silymarin. Meanwhile, the average ratio of silybin, silychristin, and silydianin in milk thistle seeds, the raw material used for silymarin extraction, was 4.3:1:0.2. This was defined as the natural ratio. Notably, the findings showed that the content of silybin decreases during extraction.

The purpose of the study was to explore the influence of the silybin, silychristin, and silydianin ratio on the bioactivity of silymarin. However, silymarin also contains other compounds, such as taxifolin and numerous unidentified chemicals, which influence its bioactivity. Therefore, in the present study, silymarin was prepared using only high‐purity silybin, silychristin, and silydianin to eliminate the interference of other impurities. Two types of formulations were prepared and studied: Silymarin containing the natural ratio of silybin, silychristin, and silydianin (4.3:1:0.2; called the natural silymarin mixture) and silymarin containing the commercial ratio of these compounds (2.5:1:0.3; called the commercial silymarin formulation).

**FIGURE 3:**
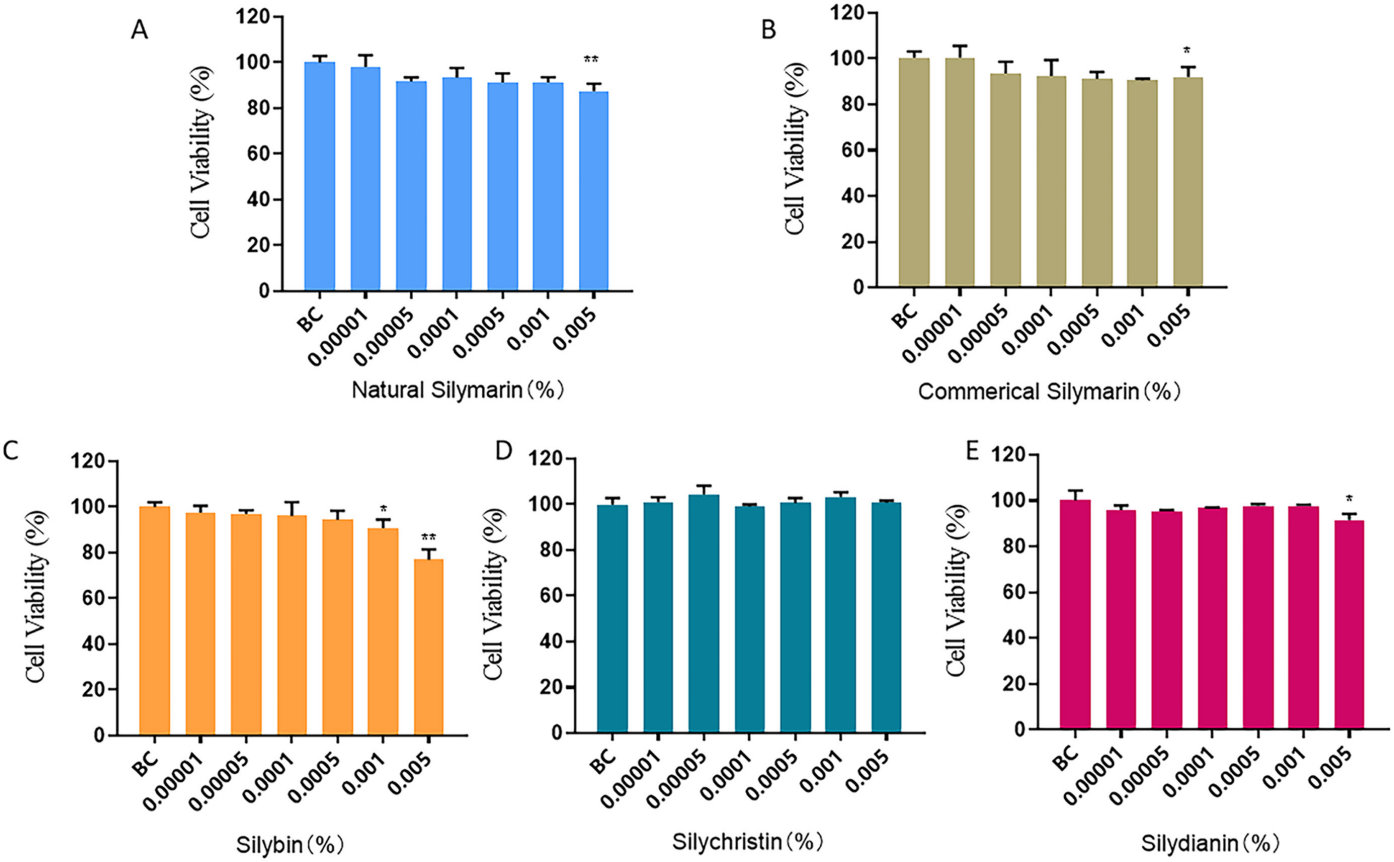
Cytotoxicity of silymarin and its components. (A) Natural silymarin mixture. (B) Commercial silymarin formulation. (C) Silybin alone. (D) Silychristin alone. (E) Silydianin alone. BC: Untreated cells. 0.00001%, 0.00005%, 0.0001%, 0.0005%, 0.001%, and 0.005% w/w of silybin, silychristin, or silydianin are equivalent to 0.207, 1.036, 2.07, 10.36, 20.7, and 103.6 μMol/L, respectively. P‐values were calculated using the two‐tailed Student's *t*‐test. (*p < 0.05, **p < 0.01 vs. BC).

The cytotoxicity of both the natural silymarin mixture (containing the natural ratio of phytochemicals) and commercial silymarin formulation (containing the commercial ratio of phytochemicals) was subsequently tested (Figure 3). Both the natural and commercial silymarin mixtures induced no significant cytotoxicity at concentrations up to 0.001%. In contrast, silybin and silydianin showed significant cytotoxicity at concentrations of 0.001% and 0.005%, respectively. However, silychristin exhibited no significant cytotoxicity even at a concentration of 0.005%. Thus, silybin showed the highest cytotoxicity among the individual flavonolignans, followed by silydianin and silychristin. Nevertheless, the viability of fibroblasts treated with natural silymarin, commercial silymarin, silybin alone, silychristin alone, and silydianin alone at a concentration of 0.005% remained higher than 70%. Therefore, 0.005% was chosen as the highest treatment concentration to test the bioactivities of silybin, silydianin, silychristin, and silymarin in the present study.

**FIGURE 4:**
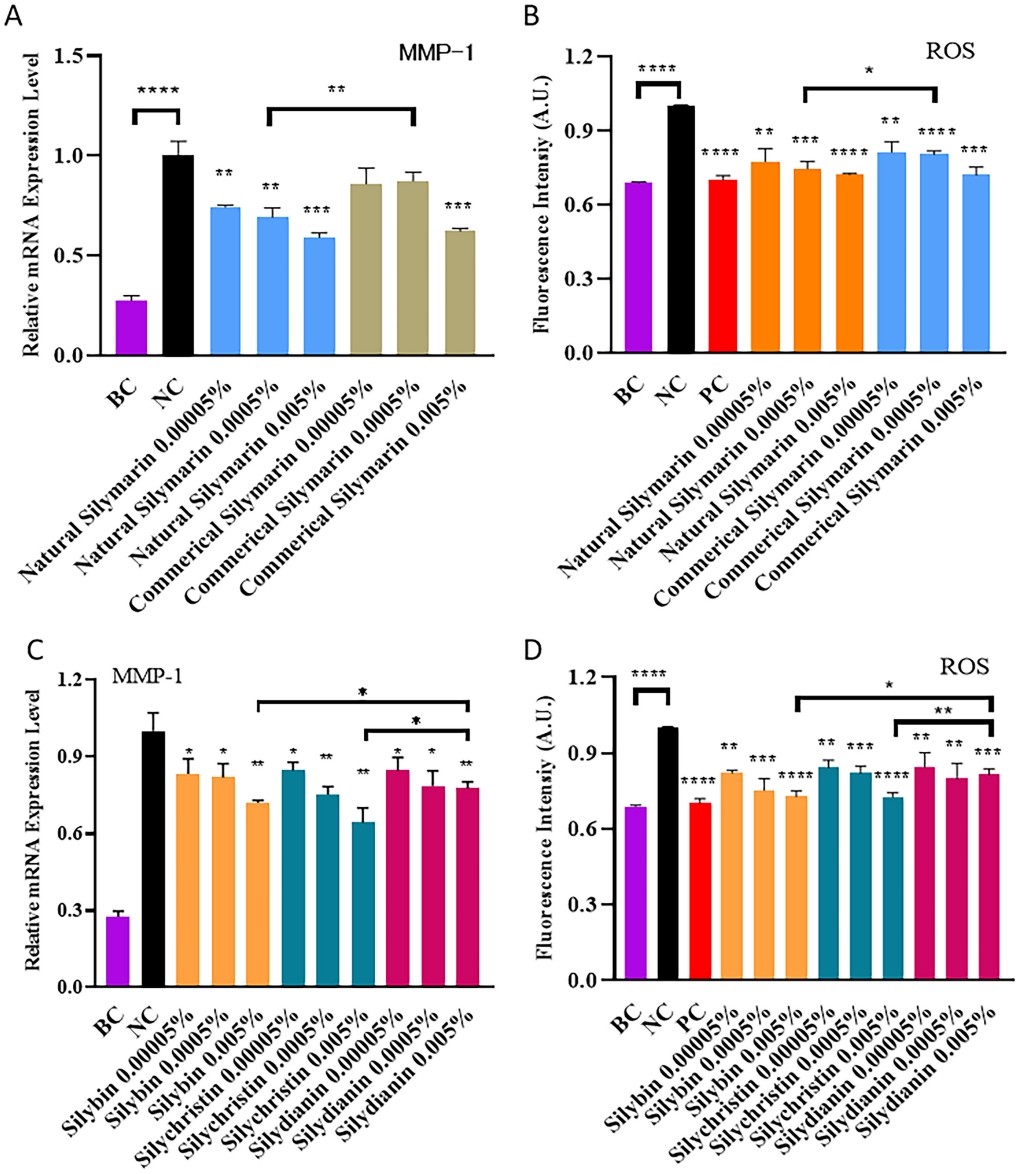
Inhibition of collagen degradation by silymarin. (A) Inhibition of MMP‐1 mRNA expression by natural and commercial silymarin mixtures. (B) Inhibition of ROS production by natural and commercial silymarin mixtures. (C) Inhibition of MMP‐1 mRNA expression by silybin, silychristin, and silydianin. (D) Inhibition of ROS production by silybin, silychristin, and silydianin. BC: Untreated cells. NC: UVA‐treated cells. PC: UVA‐ and vitamin C‐treated cells. 0.00005%, 0.0005%, and 0.005% w/w of silybin, silychristin, or silydianin are equivalent to 1.036, 10.36, and 103.6 μMol/L, respectively. P‐values were calculated using the two‐tailed Student's *t*‐test. (*p < 0.05, **p < 0.01, ***p < 0.001, and ****p < 0.0001 vs. NC, if not specified).

Collagen degradation by MMPs is considered the main mechanism of photoaging, and it induces wrinkle formation and skin sagging [7]. MMP‐1 can efficiently cleave the triple helix structure of type I and type III collagen fibers [19]. Subsequently, MMP‐3 and MMP‐9 degrade the resulting collagen fragments [19]. Silybin has been reported to inhibit the expression of MMPs and thereby counteract photoaging [20]. In this study, we compared the MMP‐1 inhibition capacity of natural silymarin and commercial silymarin (Figure 4A). Both natural and commercial silymarin showed significant dose‐dependent MMP‐1 inhibition capacities. At concentrations of 0.005% (w/w), the natural silymarin and commercial silymarin samples showed similar levels of MMP‐1 inhibition. However, when the concentration of these mixtures was lower (i.e., 0.0005% w/w), natural silymarin showed a higher MMP‐1 inhibition capacity than commercial silymarin. At high concentrations, the amount of silybin, silychristin, and silydianin in both groups of samples exceeded the amount required for maximal MMP‐1 inhibition. When sufficient amounts of silybin, silychristin, and silydianin were present, the ratio of these compounds did not affect the MMP‐1 inhibition efficiency of silymarin. However, at lower silymarin concentrations, wherein the amounts of silybin, silychristin, and silydianin were insufficient to induce maximal MMP‐1 inhibition, the ratio of these compounds affected the MMP‐1 inhibition capacity of silymarin. Overall, the results indicated that the natural silymarin mixture had better anti‐aging efficacy than the commercial silymarin formulation.

MMP generation is known to be a common consequence of ROS accumulation [9]. Thus, we explored whether the superior MMP inhibition capacity of natural silymarin over commercial silymarin can be explained by its ROS scavenging capacity (Figure 4B). Both natural and commercial silymarin mixtures showed significant dose‐dependent ROS scavenging effects. At a high concentration of 0.005% (w/w), the two types of silymarin samples showed similar ROS scavenging efficiencies. In contrast, at a lower concentration of 0.0005%, the natural silymarin mixture showed higher ROS scavenging efficiency than the commercial silymarin formulation. Hence, the trends of ROS scavenging were consistent with the MMP inhibition capacities of the natural and commercial silymarin mixtures. Notably, the silymarin mixture containing the natural ratio of phytochemicals showed better efficiency at a moderate concentration than the chemical formulation. This was likely due to the optimization of the ratio, producing superior MMP inhibition.

We examined the MMP inhibition capacities of silybin, silychristin, and silydianin alone and found that the capacity of silybin and silychristin was comparable but higher than that of silydianin (Figure 4C). To explain the differences in ROS scavenging capacity between natural and chemical silymarin, the ROS scavenging capacities of silybin, silychristin, and silydianin alone were also examined (Figure 4D). All three compounds were found to exhibit dose‐dependent ROS scavenging effects. Specifically, the ROS scavenging capacity of silybin and silychristin was comparable but higher than that of silydianin, which was in line with their MMP inhibition capacities. The result further confirmed that ROS scavenging played a key role in the MMP inhibition process of silymarin.

In commercial silymarin formulations, the concentrations of silybin, silychristin, and silydianin were 66%, 26%, and 8%, respectively. In the natural silymarin mixture, the concentrations were 78%, 18%, and 4%, respectively. Thus, natural silymarin had a higher concentration of silybin but a lower concentration of silychristin and silydianin. In addition, the total concentration of silybin and silychristin was higher in natural silymarin than in commercial silymarin. This explained why the ROS scavenging capacity of natural silymarin was higher than that of commercial silymarin.

**FIGURE 5:**
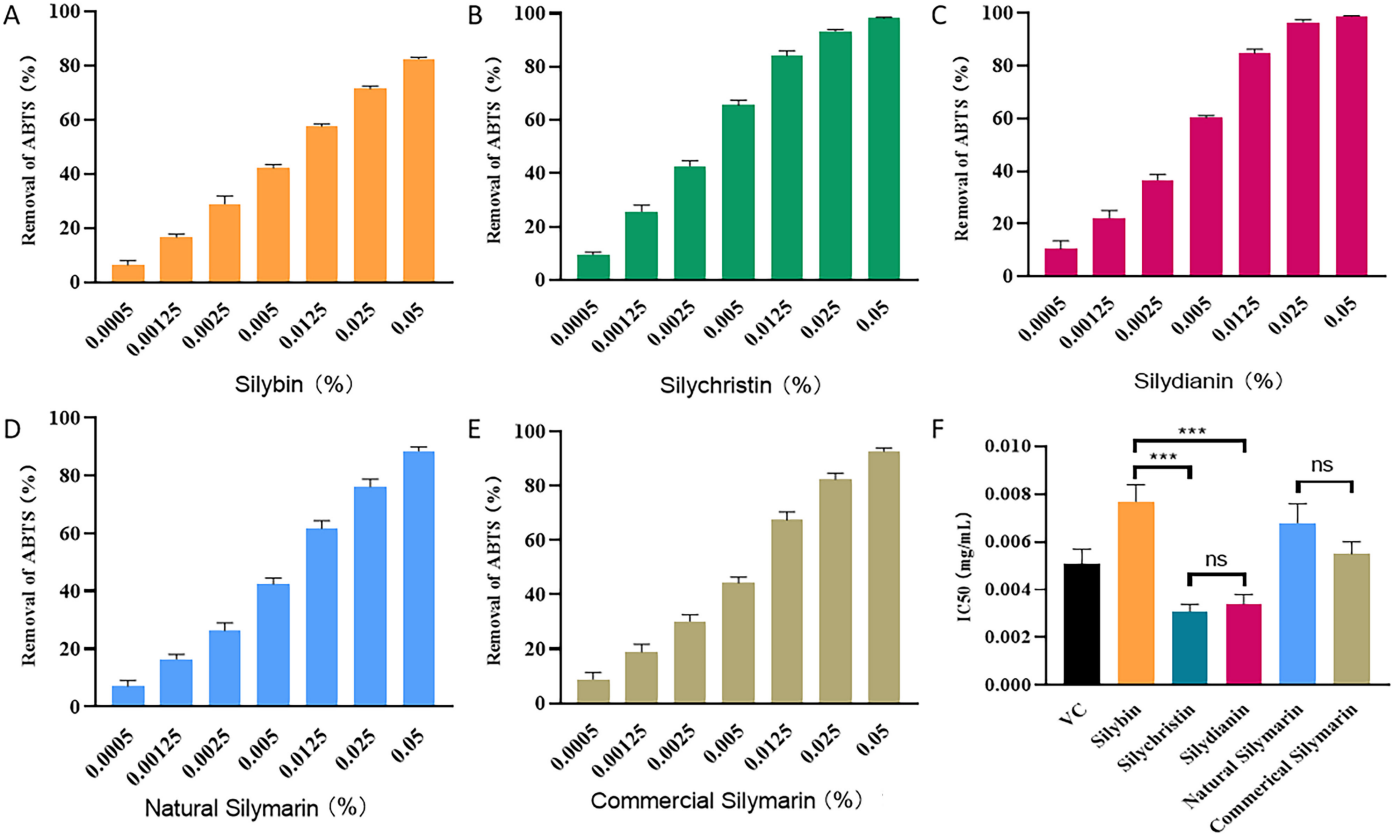
Reducing power of silymarin and its major components. (A–E) Removal of ABTS by silybin (A), silychristin (B), silydianin (C), the natural silymarin mixture (D), and the commercial silymarin mixture (E). (F) IC50 of different reagents against ABTS free radicals. 0.0005%, 0.00125%, 0.0025%, 0.005%, 0.0125%, 0.025%, and 0.05% w/w of silybin, silychristin, or silydianin are equivalent to 10.36, 25.91, 51.82, 103.6, 259.1, 518.2, and 1 036 μMol/L, respectively. VC: Vitamin C. P‐values were calculated using the two‐tailed Student's *t*‐test. (*p < 0.05, **p < 0.01, and ***p < 0.001).

The ROS scavenging capacity of compounds is often related to their reducing capacity [9]. Therefore, we studied the reducing capacity of natural silymarin, commercial silymarin, and silybin, silychristin, and silydianin alone (Figure 5). No significant difference was observed between the ABTS scavenging capacity of natural silymarin and commercial silymarin. The ABTS scavenging capacity was similar between silydianin and silychristin, but it was lower in silybin. These results were roughly consistent with previous reports, which showed that the ABTS scavenging capacity of silybin is weaker than that of silychristin and silydianin [18]. However, we did not observe any difference in the ABTS scavenging capacities of silychristin and silydianin. This contradicted the ROS scavenging data (Figure 4), indicating that the ROS scavenging effects of silymarin are mediated by mechanisms other than reducing power. Thus, we inferred that the enhanced ROS scavenging could be attributed to alternative mechanisms, such as the upregulation of antioxidant enzyme activity, in line with previous studies [21].

**FIGURE 6:**
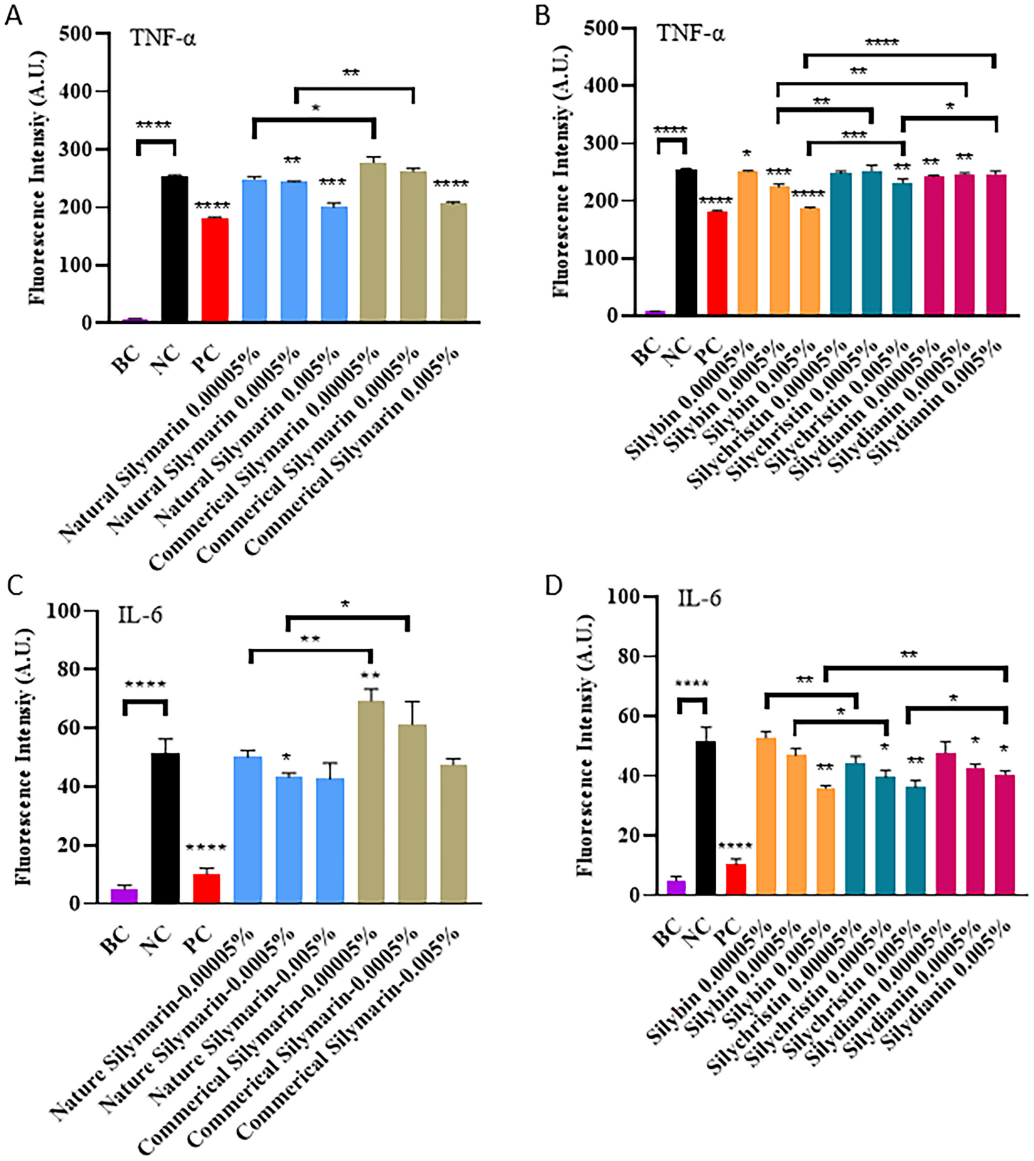
Inhibition of pro‐inflammatory factors by silymarin. (A, C) Inhibition of TNF‐α (A) and IL‐6 (C) by natural and commercial silymarin mixtures. (B, D) Inhibition of TNF‐α (A) and IL‐6 (D) by silybin, silychristin, and silydianin. BC: Untreated cells. NC: Lipopolysaccharide (LPS)‐treated cells. PC: LPS‐ and dexamethasone (DXM)‐treated cells. 0.00005%, 0.0005%, and 0.005% w/w of silybin, silychristin, or silydianin are equivalent to 1.036, 10.36, and 103.6 μMol/L, respectively. P‐values were calculated using the two‐tailed Student's *t*‐test. (*p < 0.05, **p < 0.01, ***p < 0.001, and ****p < 0.0001 vs. NC, if not specified).

Inflammation is a key factor causing skin aging [22]. Pro‐inflammatory factors such as TNF‐α and IL‐6 are significantly upregulated following LPS stimulation (Figure 6). In the present study, compared to commercial silymarin, natural silymarin was found to be more efficient at attenuating TNF‐α levels, especially at a concentration of 0.0005%. At lower concentrations, however, its TNF‐α inhibition effect was not obvious. Meanwhile, at higher concentrations, both natural and commercial silymarin provided similar levels of TNF‐α inhibition. With regard to IL‐6, the inhibition capacity of natural silymarin was similar to that of commercial silymarin, and no inhibitory effect was detected when the silymarin concentration was lower than 0.0005%.

In addition, the TNF‐α and IL‐6 inhibition capacities of silybin, silychristin, and silydianin were also assessed. Of these compounds, silybin showed the highest TNF‐α inhibition capacity, followed by silychristin and silydianin. This hierarchy explained why natural silymarin, which contains more silybin and less silychristin and silydianin, was more effective at inhibiting TNF‐α than commercial silymarin. Meanwhile, the IL‐6 inhibition capacities of silydianin and silychristin were similar. Although these values were higher than the IL‐6 inhibition capacity of silybin, the difference was not significant. This was likely the reason for the negligible difference in IL‐6 inhibitory effects between natural and commercial silymarin formulations.

**FIGURE 7:**
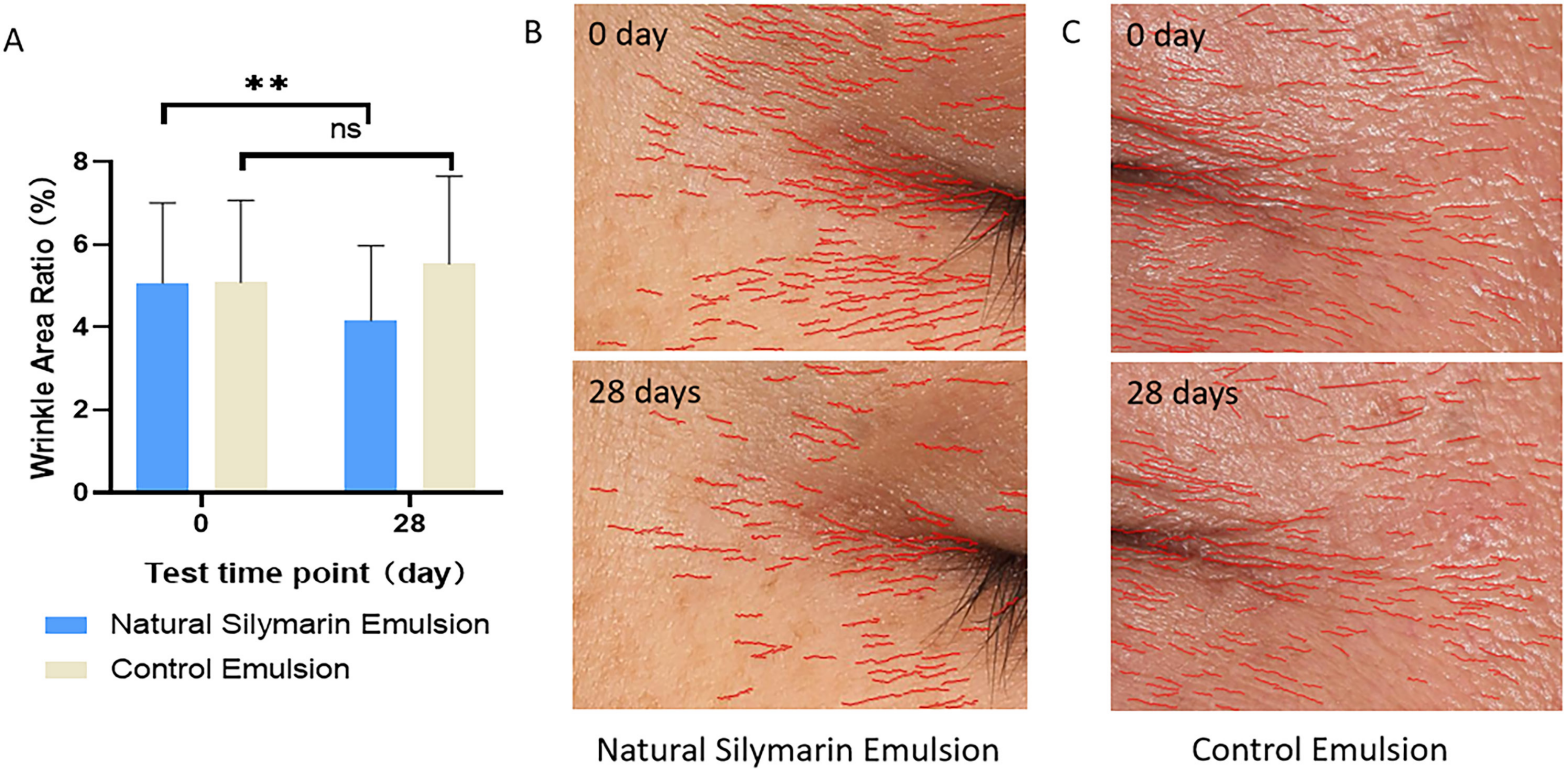
Anti‐wrinkle effects of the natural silymarin mixture. (A) Wrinkle area ratio before and after natural treatment with the natural silymarin mixture. (B, C) Representative images of the skin before and after treatment with the natural silymarin emulsion (B) and the control emulsion (C). P‐values were calculated using the two‐tailed Student's *t*‐test. (*p < 0.05 and **p < 0.01, *n* = 30).

Owing to its excellent capacity for MMP inhibition, ROS scavenging, and pro‐inflammatory factor attenuation, natural silymarin appeared to be a good anti‐aging agent. Thus, we tested its anti‐wrinkle effects in a cohort of 30 volunteers (Figure 7). Since silymarin could not be directly applied to the skin, we prepared a simple emulsion containing the natural silymarin mixture, without any other bioactive ingredients. After 28 days, the wrinkle area was found to decrease considerably on the side of the face treated with the natural silymarin emulsion. Meanwhile, no such changes were observed on the other half of the face, which was treated with a control emulsion, without natural silymarin. These results indicated that natural silymarin was effective at decreasing wrinkles.

It should be noted that the “optimal ratio” of phytochemicals in silymarin is particularly relevant when the concentration of silymarin falls within the moderate range. At high concentrations, even suboptimal ratios can provide sufficient silybin, silychristin, and silydianin. As a result, the advantages of the optimal ratio become less apparent. Meanwhile, when the concentration of silymarin is too low, the amounts of silybin, silychristin, and silydianin are insufficient to achieve anti‐aging effects, even when the ratios of these compounds are close to optimal.

Notably, this study reports that the natural silymarin formulation offers stronger anti‐aging effects than commercial silymarin formulations. However, both these agents are effective at inhibiting MMP production, scavenging ROS, and inhibiting pro‐inflammatory factors. The difference between their activities is significant but not enormous; the gap lies in a small difference of 12% silybin, 8% silychristin, and 4% silydianin.

On a practical level, the concentration of silymarin used in cosmetic products is often lower than adequate. First, regulatory guidelines restrict the maximum allowable dosage of silymarin. Second, both the contact time and the amount of silymarin delivered to the skin are inherently limited when topical formulations are used. Finally, part of the silymarin may not penetrate the layers of the skin following topical application. Hence, the use of silymarin with an optimal ratio of phytochemicals may be helpful for achieving better anti‐aging efficacy.

## 4 Conclusion

This study shows that the ratio of silybin, silychristin, and silydianin—the three main components of silymarin—differs between natural silymarin (milk thistle seeds) and commercial formulations. Owing to this, the MMP inhibition capacity, ROS scavenging ability, and TNF‐α‐downregulating effects of silymarin containing a natural phytochemical ratio are better than those of commercial silymarin formulations. These differences are due to the variations in MMP inhibition, ROS scavenging, and anti‐inflammatory properties among silybin, silychristin, and silydianin. The natural silymarin mixture contains more silybin but less silychristin and silydianin than commercial formulations. Its MMP inhibition capacity can be attributed to the ROS scavenging capacity of its components, which is similar between silybin and silychristin but lower in silydianin. However, the ROS scavenging capacity of this mixture cannot be fully attributed to its reducing power. The TNF‐α inhibition capacity is the highest in silybin, followed by silychristin and silydianin. However, silybin, silychristin, and silydianin show comparable IL‐6 inhibition activities; therefore, the IL‐6 inhibition capacity of natural and commercial silymarin formulations is also comparable. Nevertheless, our findings demonstrate that natural silybin is a satisfactory anti‐aging ingredient and can produce anti‐wrinkle effects on facial skin in humans. These findings demonstrate its value as a functional ingredient in cosmetic products.

## Author Contributions

Sen Hou conceived the study, drafted the manuscript, and supervised the entire project. Anning Wang and Hanliang Song conducted the experiments and analyzed the data. Anzhang Li provided critical suggestions for improvement and supervised the entire project. The final version of the manuscript was approved by all authors.

## Funding

The authors have nothing to report.

## Ethics Statement

This study received ethics approval from the Institutional Review Board of Guangzhou Zhongkejian Technology Testing Company Ltd. (IRB202506‐001). All experimental protocols adhered to the Declaration of Helsinki.

## Consent

All participants provided written informed consent prior to their inclusion in the study, following full disclosure of the research aims, procedures, and potential risks.

## Conflicts of Interest

The authors declare no conflicts of interest.

## Supporting information


**Table S1:** Formula of sebum containing natural silymarin.
**Figure S1:** Removal of ABTS by vitamin C.

## Data Availability

The data that support the findings of this study are available from the corresponding author upon reasonable request.

## References

[1] Y. Yanyin , Y. Zhiguo , H. Shiguo , L. Yan , X. Qiang , and S. Huasu , “The Efficacy of Silymarin in Dermatology and Its Application in Cosmetics,” China Surfactant Detergent & Cosmetics 49 (2019): 259–263.

[2] J. I. Lee , B. H. Hsu , D. Wu , and J. S. Barrett , “Separation and Characterization of Silybin, Isosilybin, Silydianin and Silychristin in Milk Thistle Extract by Liquid Chromatography‐Electrospray Tandem Mass Spectrometry,” Journal of Chromatography A 1116 (2006): 57–68. 16631762 10.1016/j.chroma.2006.03.053

[3] V. Simanek , V. Kren , J. Ulrichova , J. Vicar , and L. Cvak , “Silymarin: What Is in the Name? An Appeal for a Change of Editorial Policy,” Hepatology 32 (2000): 442–444. 10960282 10.1053/jhep.2000.9770

[4] F. Liu and S. J. Pang , “Stress Tolerance and Antioxidant Enzymatic Activities in the Metabolisms of the Reactive Oxygen Species in Two Intertidal Red Algae Grateloupia Turuturu and *Palmaria Palmata* ,” Journal of Experimental Marine Biology and Ecology 382 (2010): 82–87.

[5] I. R. Davison and G. A. Pearson , “Stress Tolerance in Intertidal Seaweeds,” Journal of Phycology 32 (2010): 197–211.

[6] M. J. Dring , “Stress Resistance and Disease Resistance in Seaweeds: The Role of Reactive Oxygen Metabolism,” Advances in Botanical Research 43 (2005): 175–207 New York Ny, Elsevier.

[7] P. Agin , “Photoaging/Photodamage and Photoprotection,” Journal of the American Academy of Dermatology 24 (1991): 315–317. 2007693 10.1016/s0190-9622(08)80629-9

[8] T. G. Polefka , T. A. Meyer , P. P. Agin , and R. J. Bianchini , “Effects of Solar Radiation on the Skin,” Journal of Cosmetic Dermatology 11 (2012): 134–143. 22672278 10.1111/j.1473-2165.2012.00614.x

[9] K. K. Dong , N. Damaghi , S. D. Picart , N. G. Markova , and D. B. Yarosh , “UV‐Induced DNA Damage Initiates Release of MMP‐1 in Human Skin,” Experimental Dermatology 17 (2008): 1037–1044. 18459971 10.1111/j.1600-0625.2008.00747.x

[10] J. Ryu , S. J. Park , I. H. Kim , Y. Choi , and T. J. Nam , “Protective Effect of Porphyra‐334 on UVA‐Induced Photoaging in Human Skin Fibroblasts,” International Journal of Molecular Medicine 34 (2014): 796–803. 24946848 10.3892/ijmm.2014.1815 PMC4121349

[11] A. Svobodova , A. Zdarilova , D. Walterova , and J. Vostalova , “Flavonolignans From *Silybum marianum* Moderate UVA‐Induced Oxidative Damage to HaCaT Keratinocytes,” Journal of Dermatological Science 48 (2007): 213–224. 17689055 10.1016/j.jdermsci.2007.06.008

[12] J. Sonnenbichler and I. Zetl , “Biochemical Effects of the Flavonolignane Silibinin on RNA, Protein and DNA Synthesis in Rat Livers,” Progress in Clinical and Biological Research 213 (1986): 319–331. 2424029

[13] C. Loguercio and D. Festi , “Silybin and the Liver: From Basic Research to Clinical Practice,” World Journal of Gastroenterology 17 (2011): 2288–2301. 21633595 10.3748/wjg.v17.i18.2288 PMC3098397

[14] S. Kitajima and K. Yamaguchi , “Silybin From *Silybum Marianum* Seeds Inhibits Confluent‐Induced Keratinocytes Differentiation as Effectively as Retinoic Acid Without Inducing Inflammatory Cytokine,” Journal of Clinical Biochemistry and Nutrition 45 (2009): 178–184. 19794926 10.3164/jcbn.09-20 PMC2735630

[15] M. Reina and A. Martinez , “Is Silybin the Best Free Radical Scavenger Compound in Silymarin?,” Journal of Physical Chemistry B 120 (2016): 4568–4578. 27149000 10.1021/acs.jpcb.6b02807

[16] N. C. Kim , T. N. Graf , C. M. Sparacino , M. C. Wani , and M. E. Wall , “Complete Isolation and Characterization of Silybins and Isosilybins From Milk Thistle (*Silybum Marianum*),” Organic & Biomolecular Chemistry 1 (2003): 1684–1689. 12926355 10.1039/b300099k

[17] L. Abenavoli , R. Capasso , N. Milic , and F. Capasso , “Milk Thistle in Liver Diseases: Past, Present, Future,” Phytotherapy Research 24 (2010): 1423–1432. 20564545 10.1002/ptr.3207

[18] M. Pyszkova , M. Biler , D. Biedermann , et al., “Flavonolignan 2,3‐Dehydroderivatives: Preparation, Antiradical and Cytoprotective Activity,” Free Radical Biology & Medicine 90 (2016): 114–125. 26582372 10.1016/j.freeradbiomed.2015.11.014

[19] T. Quan and G. J. Fisher , “Role of Age‐Associated Alterations of the Dermal Extracellular Matrix Microenvironment in Human Skin Aging: A Mini‐Review,” Gerontology 61 (2015): 427–434. 25660807 10.1159/000371708 PMC4524793

[20] B. Anfuso , P. Giraudi , C. Tiribelli , and N. Rosso , “Silybin Modulates Collagen Turnover in an in Vitro Model of NASH,” Molecules 24 (2019), 10.3390/molecules24071280. PMC6479571 30986937

[21] Y. Fu , C. Guo , H. Wu , and C. Chen , “Arginine Decarboxylase ADC2 Enhances Salt Tolerance Through Increasing ROS Scavenging Enzyme Activity in *Arabidopsis thaliana* ,” Plant Growth Regulation 83 (2017): 253–263.

[22] B. A. Gilchrest , J. S. Stoff , and N. A. Soter , “Chronologic Aging Alters the Response to Ultraviolet‐Induced Inflammation in Human Skin,” Journal of Investigative Dermatology 79 (1982): 11–15. 6953155 10.1111/1523-1747.ep12510417

