# Supplementary material for: Enhanced Anti‐Aging Properties of Silymarin With a Natural Phytochemical Ratio Over Commercial Formulations
Source: J Cosmet Dermatol. 2026 Mar 5;25(3):e70780. doi: 10.1111/jocd.70780 (PMC12963528; doi:10.1111/jocd.70780)
Supplement: Supplementary file 1 — Table S1: Formula of sebum containing natural silymarin. Figure S1: Removal of ABTS by vitamin C. [file JOCD-25-e70780-s001.docx]

Supporting Information

Better Anti-aging Property of Silymarin with Natural Ratio over Commercial Ratio

**Table of Contents**

Table S1: Formula of sebum containing natural silymarin.

Figure S1: Removal of ABTS by vitamin C.

Table S1: Formula of sebum containing natural silymarin.

| Group | Ingredients | Ratio (w/w) % | Procedure |
| --- | --- | --- | --- |
| A | Water | 79.77 | 1. B was heated to 85 ^o^C. 2. D was mixed and heated to 60-65 ^o^C. 3. E was mixed sufficiently. 4. A was heated to 85 ^o^C and mixed. 5. B was added and mixed at 3000 rpm for 3min. 6. C was added and mixed for 2 min. 7. The mixture was cooled down to 45 ^o^C. 8. D and E were added and mixed. |
|  | Disodium EDTA | 0.03 |  |
|  | Ammonium Acryloyldimethyl Taurate/VP Copolymer | 0.35 |  |
|  | Butylene Glycol | 4 |  |
| B | C14-22 Alcohols (80%), C12-20 Alkyl Glucoside (20%) | 1.5 |  |
|  | Isononyl Isononanoate | 3 |  |
|  | Caprylic/Capric Triglyceride | 3 |  |
| C | Polyacrylamide (40%), Water (30%), C13-14 Isoparaffin (24%) Laureth-7 (6%) | 0.25 |  |
| D | Hydroxyacetophenone | 0.4 |  |
|  | Butylene Glycol | 2 |  |
|  | 1,2-Hexanediol | 0.5 |  |
| E | Silymarin (natural ratio) | 0.1 |  |
|  | Xanthan Gum | 0.1 |  |
|  | Butylene Glycol | 1 |  |
|  | Water (70 ^o^C) | 4 |  |


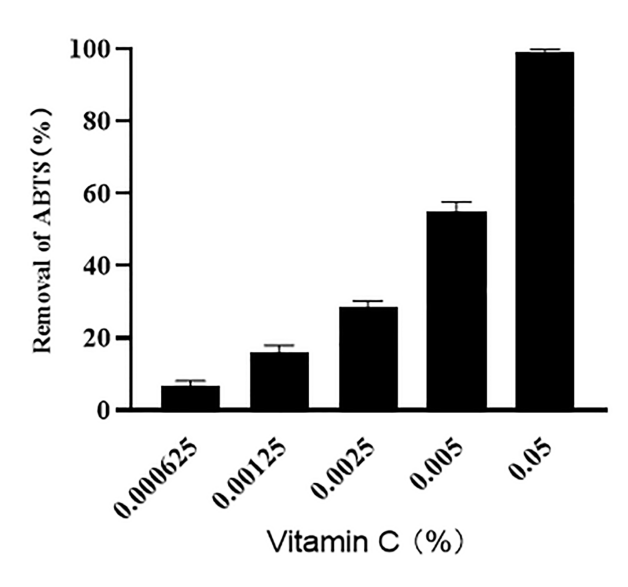


Figure S1: Removal of ABTS by vitamin C.
